# The relationship between tobacco and non-alcoholic fatty liver disease incidence: a systematic review and meta-analysis of observational studies

**DOI:** 10.3389/fmed.2025.1670932

**Published:** 2025-10-15

**Authors:** Jianxiang Jin, Yuping Zhang, Yiping Huang

**Affiliations:** Affiliated Jinhua Hospital, Zhejiang University School of Medicine, Jinhua, Zhejiang, China

**Keywords:** NAFLD, active smoking, passive smoking, incidence, meta-analysis

## Abstract

**Background:**

This meta-analysis investigates the relationship between smoking and non-alcoholic fatty liver disease (NAFLD) risk.

**Methods:**

Observational studies (cohort, case-control, cross-sectional) were systematically searched in PubMed, Web of Science, EBSCO, and Cochrane Library up to December 2024. Adjusted odds ratio (OR) and corresponding 95% confidence interval (95% CI) were used to assess the association.

**Results:**

A total of 19 studies, composing 450,130 participants were included. Active smoking significantly increased NAFLD risk (OR = 1.30, 95% CI: 1.21–1.40, *p* < 0.001), with stronger effects observed in current smokers (OR = 1.41, 95% CI: 1.22–1.63, *p* < 0.001). A dose-response relationship was evident: ≥20 pack-years of smoking elevated risk by 32% (OR = 1.32, 95% CI: 1.18–1.49, *p* < 0.001). Subgroup analyses revealed amplified risks in metabolically compromised individuals, including those with BMI ≥ 24 (OR = 1.43, *p* < 0.001), TG ≥ 1.2 mmol/L (OR = 1.41, *p* = 0.003), and SBP ≥ 125 mmHg (OR = 1.65, *p* < 0.001). Passive smoking showed a marginal association (OR = 1.13, 95% CI: 1.09–1.16, *p* < 0.001).

**Conclusion:**

Smoking is an independent risk factor for NAFLD, particularly in individuals with metabolic dysregulation. Public health strategies targeting smoking cessation and metabolic control may mitigate NAFLD burden.

**Systematic review registration:**

https://www.crd.york.ac.uk/prospero/#loginpage, identifier CRD42024545970

## Introduction

Non-alcoholic fatty liver disease (NAFLD) is one of the most prevalent chronic liver diseases, with an estimated global prevalence of 25% (1). It poses significant health risks, increasing the incidence of metabolic syndrome, type 2 diabetes mellitus, and cardiovascular events (2). The all-cause mortality rate of NAFLD patients is significantly higher than that of the general population, mainly due to cardiovascular diseases and extrahepatic malignancies (3). Moreover, NAFLD has also been linked to a higher risk of osteoporosis, chronic kidney disease, colorectal cancer, and breast cancer (4, 5). Prevention strategies for NAFLD include maintaining a healthy lifestyle, such as a balanced diet, regular exercise, and weight control, as well as managing underlying metabolic disorders like obesity, hypertension, and diabetes (6, 7).

Several common risk factors contribute to the development of NAFLD, including obesity, insulin resistance, and a sedentary lifestyle (8). Among these, smoking has emerged as a potential risk factor. Smoking may exacerbate the severity of NAFLD through mechanisms such as inducing oxidative stress, promoting hepatocyte apoptosis, and stimulating the extracellular signal-regulated kinase signaling pathway (9–11). It has been suggested that smoking can increase the risk of NAFLD, with a dose-response relationship observed between the amount of smoking and the risk of developing the disease (12). Furthermore, emerging evidence suggests that the association between smoking and NAFLD may be modified by metabolic factors, such as obesity or insulin resistance, indicating a potential synergistic effect that warrants further investigation. However, the exact role of smoking in the development and progression of NAFLD remains a subject of debate.

Numerous studies have investigated the impact of smoking on NAFLD, yielding conflicting results. Firstly, some studies such as Yun Seo Jang et al. (13) and Ayaka Hamabe et al. (14) did not observe a link between smoking and NAFLD risk. Secondly, subgroup analyses of smoking frequency, duration of quitting smoking, and other factors in different studies show a high degree of inconsistency. For example, Ayaka Hamabe et al. (14) believe that smoking <10 pack years increases the risk of NAFLD, but Peiyi Liu et al. (15) believe that smoking <10 pack years does not increase the risk of NAFLD. These discrepancies may arise from variations in study design, population characteristics, adjustments for confounding factors, or differences in NAFLD diagnostic criteria. Given the conflicting evidence in existing literature, this study aims to systematically review and meta-analyze observational studies assessing the association between smoking and NAFLD. The goal is to provide robust evidence for smoking-related NAFLD risk and inform prevention strategies in healthy populations.

## Materials and methods

### Search strategy

In the electronic databases of PubMed, Web of science, EBSCO and the Cochrane Library, a comprehensive literature search was performed by retrieving the keywords “non-alcoholic fatty liver disease” and “smoking” until December 2024. The complete retrieval formula that was used to identify the related studies includes: (“non-alcoholic fatty liver disease” OR “non-alcoholic fatty liver disease” OR “NAFLD” OR “non-alcoholic fatty liver disease” OR “non-alcoholic fatty liver” OR “non-alcoholic fatty livers” OR “non-alcoholic steatohepatitis” OR “non-alcoholic steatohepatitides”) AND (“smoking” OR “tobacco smoke pollution” OR “tobacco use” OR “tobacco products” OR “active smoking” OR “passive smoking” OR “secondhand smoking” OR “tobacco”). Retrieved studies and recently reviewed reference lists were also reviewed for potentially inclusive studies. In cases of duplicate publication, the original article is included if the study is published as an abstract and an original article. Also, if several articles were published for research, only the latest or highest quality articles were included. This meta-analysis was conducted according to the Meta-Analysis of Observational Studies in Epidemiology (MOOSE) guidelines (16). The population, intervention/exposure, comparison, outcome, and setting (PICOS) criteria were used to describe the research question. The Prospero registration number of this meta-analysis was CRD42024545970.

### Selection criteria

An eligible criterion had been formulated. The specific criteria were as follows: inclusion criteria: (1) all included studies are observational studies (include prospective or retrospective cohort studies, case-control studies, and cross-sectional studies). (2) The main exposure of study was smoking including active and passive smoking, and the outcome was NAFLD risk. (3) The article must report extractable risk estimates (e.g., RR, HR, OR) along with their 95% confidence intervals, or provide raw data sufficient to calculate these estimates. Exclusion criteria: (1) the study was conducted on NAFLD population. (2) The study was published in duplicate. (3) The study was not published in English. (4) The study was case reports, case series, reviews, editorials, conference abstracts (without full data), and animal or *in vitro* studies.

Two authors independently applied a search strategy to select studies from the database and independently reviewed the titles and abstracts of these articles to determine eligibility for inclusion. When in doubt, the full text will be searched for further selection. When necessary, the authors were contacted for more information about their research. In case of disagreement, discussions were conducted with the third author. When consensus could not be reached, the study was excluded.

### Data collection and quality assessment

A jointly agreed data collection form was used to extract all data. Information was extracted as follows: the author’s name, year of publication, study type, age, exposure assessment, number of participants, number of NAFLD cases, number of smokers, number of non-smokers, variables adjusted in the statistical analyses, and outcomes. To ensure the objectivity and accuracy of the data, two researchers independently extracted data from each study. Disagreements were resolved by consensus or consultation with a third author.

The quality of each cohort and case-control study was evaluated by the Newcastle-Ottawa Quality Assessment Scale (NOS) checklist, a tool used for quality assessment of non-randomized studies. NOS checklist is composed of eight items classified into three aspects, including selection, comparability, and outcome. The maximum scores of this checklist were nine, and scores between seven and nine were identified to higher study quality. The cross-sectional studies were assessed by the Agency for Healthcare Research and Quality (AHRQ) Recommended Standard List. Risk of bias was assessed as “low risk,” “high risk” or “unclear risk.” To ensure the reliability and standardization of the quality assessment, the evaluation was also independently performed by two researchers. Any discrepancies were resolved through discussion with a third researcher.

### Objectives and endpoints

The primary objective is to explore the relationship between smoking and the incidence of NAFLD. The secondary objective was to explore the relationship between the incidence of NAFLD and the smoking subgroup, such as smoking duration, smoking intensity, pack-years smoked and other metabolic indicators. The results after adjusting for relevant confounding factors were uniformly adopted for the processing of relevant data from the included articles.

### Data analysis

The Stata software version 12 (StataCorp, College Station, Texas, USA) was used to analyze the data. The confidence interval (CI) of odds ratio (OR) was set at 95% to examine the relationship between smoking and BC risk. Heterogeneity of included studies was tested by Q statistic and I^2^ statistic to quantitatively assess inconsistency. For statistical results, values of *p* < 0.10 and I^2^ > 50% were representative of statistically significant heterogeneity. To increase the credibility of the results, random effect model was uniformly adopted in this study. When more than ten studies were included, sensitivity analysis and publication bias test were performed to evaluate the stability and reliability of the results. Publication bias was evaluated by the Begg’s test. *P*-values less than 0.05 were considered statistically significant.

## Results

### Study characteristics

**FIGURE 1:**
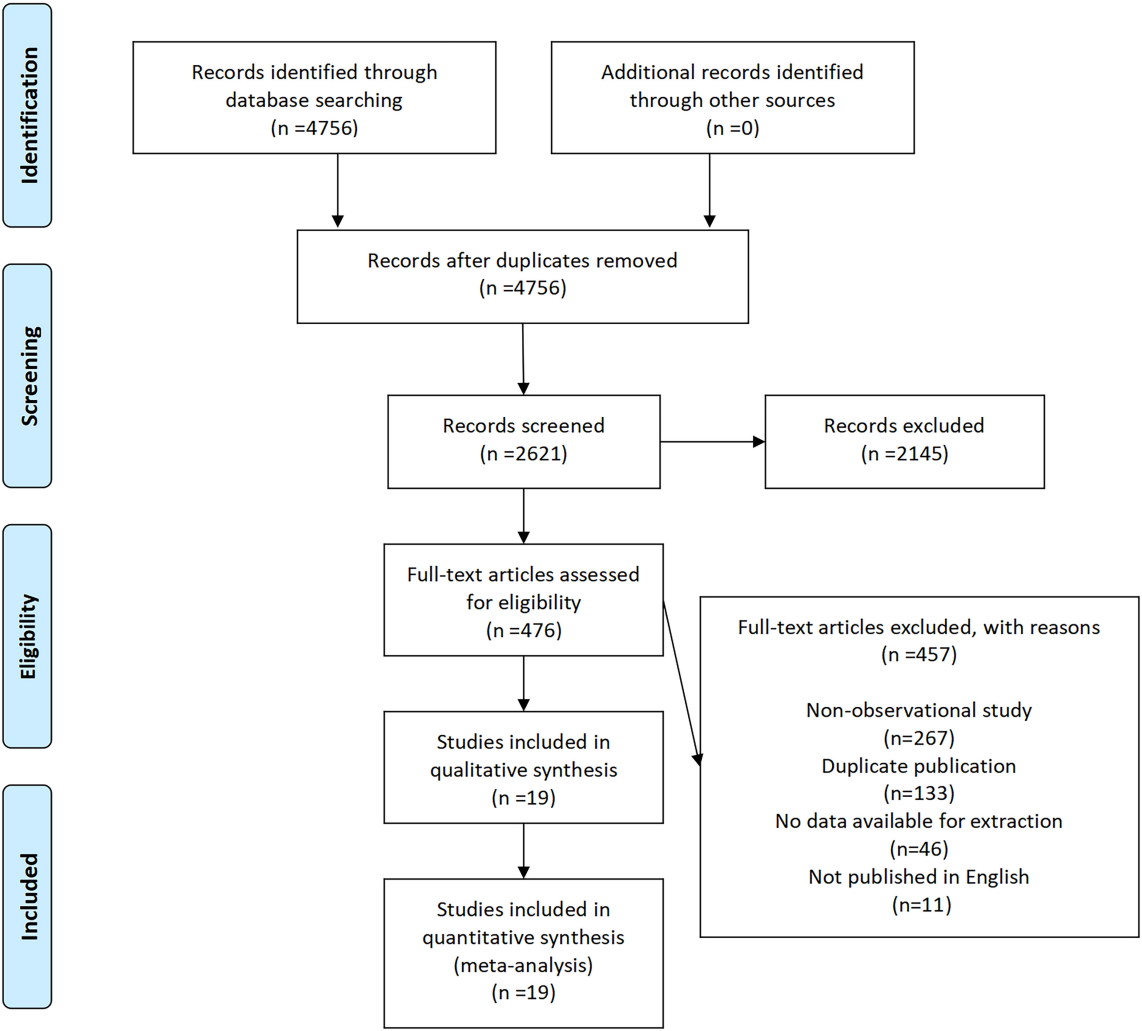
Flow chart of study selection.

A total of 4,756 records were initially identified through database searching, with no additional records obtained from other sources (Figure 1). After removing duplicates, 2,621 records remained for title and abstract screening. Of these, 2,145 records were excluded due to irrelevance. The full texts of 476 articles were assessed for eligibility, among which 457 were excluded for the following reasons: non-observational study (*n* = 219), duplicate publication (*n* = 130), no data available for extraction (*n* = 96), and not published in English (*n* = 11). Ultimately, 19 studies (10 cohort, 8 cross-sectional, 1 case-control) spanning Asia, Europe, and South America met the inclusion criteria and were included in the quantitative synthesis (Supplementary Table 1). Cohort and case-control studies demonstrated high quality (Cohort studies: mean NOS = 7.9; case-control study: NOS = 9; Supplementary Tables 2, 3), while cross-sectional studies were assessed on AHRQ criteria (Supplementary Table 4). According to the quality evaluation results of the investigators, all included studies were of high quality.

### Quality appraisal of included studies

The characteristics of the included studies are summarized in Table 1. These studies were conducted across seven countries, encompassing 450,130 participants and 81,445 cases of NAFLD, with participant recruitment periods ranging from 1980 to 2022. Among the studies, 10 were cohort studies, 8 were cross-sectional studies, and 1 was a case-control study. The median age at recruitment ranged from 32.6 to 76.4 years, with most studies involving adults aged over 40 years.

**TABLE 1:** Characteristics of included observational studies in the meta-analysis.

Author, year	Country	Time of experiment (year)	Age at recruitment (year)	Age (median year)	No. of participants	No. of NAFLD cases	Characteristics
Llorenc and Caballerı′a (44)	Spain	2007–2008	17–83	53.0	766	198	Cross-sectional study
Hamabe et al. (14)	Japan	1998–2008	NA	50.3	2029	565	Cohort study
Koehler (45)	Netherlands	2009–2012	65.3–98.7	76.4	2811	986	Cohort study
Yu Liu (46)	China	2012	>40	NA	9550	2844	Cross-sectional study
van den Berg (47)	Netherlands	2006–2013	18–91	44.0	37496	8259	Cross-sectional study
Liu et al. (15)	China	2008–2010	NA	65.3	9432	3169	Cross-sectional study
Nam Hee Kim (48)	Korea	2011–2015	NA	36.1	160862	40964	Cross-sectional study
Masashi Okamoto (49)	Japan	2003–2013	18–80	NA	3860	443	Cohort study
Xianghai Zhou (50)	China	2012–2014	26–76	49.5	3166	716	Cross-sectional study
Hsing (51)	China	2016–2018	35–85	53.6	3589	476	Cross-sectional study
Haruka Takenaka (52)	Japan	2016–2020	30–80	51.5	13466	5169	Case-control study
Wu et al. (23)	Finland	1980–2018	>18	41.3	1315	214	Cohort study
Moon (53)	Korea	2008–2015	35–75	50.6	28060	6488	Cohort study
Jeong et al. (37)	Korea	2002–2022	NA	57.0	139180	2393	Cohort study
Hu (54)	Japan	2004–2015	NA	43.5	14251	2507	Cross-sectional study
Paulina Pettinelli (55)	Chile	2016–2020	21–75	NA	2774	1169	Cohort study
Minjung Han (56)	Korea	2016–2020	>20	53.4	7096	2046	Cohort study
Jang et al. (13)	Korea	2019–2020	>19	51.7	9603	2711	Cohort study
Ying Che (57)	China	2021–2022	NA	32.6	824	128	Cohort study

NA, not available; NAFLD, non-alcoholic fatty liver disease.

### Smoking status and NAFLD

The pooled analysis demonstrated that active smoking was significantly associated with increased risk of NAFLD (OR = 1.30, 95% CI: 1.21–1.40, *p* < 0.001), with substantial heterogeneity observed (I^2^ = 94.1%). Subgroup analysis by study design revealed consistent findings: both cohort studies (OR = 1.27, 95% CI: 1.15–1.41, *p* < 0.001) and cross-sectional studies (OR = 1.32, 95% CI: 1.16–1.50, *p* < 0.001) supported this association (Table 2).

**TABLE 2:** Effects of smoking on NAFLD incidence.

Subgroup analysis	No. of studies	OR	95% CI	*P*	Heterogeneity (I^2^) (%)
Active smoking	19	1.30	1.21–1.40	<*0.001*	94.1
Cohort study	10	1.27	1.15–1.41	<*0.001*	81.5
Cross-sectional study	8	1.32	1.16–1.50	<*0.001*	95.1
Ever smoking	9	1.16	1.06–1.28	*0.002*	75.8
Cohort study	8	1.18	1.06–1.30	*0.002*	78.8
Former smoking	12	1.30	1.05–1.60	*0.015*	94.5
Cohort study	4	1.28	0.96–1.71	0.089	68.4
Cross-sectional study	8	1.30	1.00–1.70	*0.050*	96.3
Current smoking	13	1.41	1.22–1.63	<*0.001*	91.8
Cohort study	4	1.48	1.25–1.75	<*0.001*	19.1
Cross-sectional study	9	1.37	1.15–1.63	*0.001*	94.2
**TC**					
<5 mmol/L	5	1.17	0.98–1.39	0.080	81.5
≥5 mmol/L	9	1.47	1.15–1.90	*0.003*	95.0
**TG**					
<1.2 mmol/L	6	1.28	1.11–1.48	*0.001*	74.0
≥1.2 mmol/L	8	1.41	1.13–1.77	*0.003*	93.5
**HDL-C**					
<1.30 mmol/L	5	1.52	1.20–1.91	<*0.001*	95.4
≥1.30 mmol/L	5	1.11	0.95–1.31	0.188	57.3
**LDL-C**					
<3 mmol/L	3	1.12	1.00–1.25	0.054	0
≥3 mmol/L	4	1.21	0.96–1.51	0.102	92.9
**FPG**					
<5.25 mmol/L	5	1.33	0.94–1.86	0.106	97.0
≥5.25 mmol/L	6	1.40	1.18–1.66	<*0.001*	71.5
**SBP**					
<125 mmHg	7	1.07	0.97–1.18	0.152	33.7
≥125 mmHg	5	1.65	1.41–1.93	<*0.001*	74.8
**DBP**					
<75 mmHg	5	1.12	1.00–1.25	0.054	0
≥75 mmHg	3	1.50	1.05–2.14	*0.025*	97.4
**BMI**					
<23.9	10	1.21	1.08–1.34	*0.001*	87.8
Cohort study	4	1.07	0.98–1.17	0.110	57.6
Cross-sectional study	6	1.27	1.10–1.47	*0.001*	73.0
≥24	10	1.43	1.20–1.71	<*0.001*	97.1
Cohort study	4	1.58	1.35–1.85	<*0.001*	9.1
Cross-sectional study	6	1.38	1.11–1.71	*0.004*	98.3
**Age**					
<50 years	12	1.27	1.08–1.48	*0.003*	96.7
Cohort study	4	1.14	0.97–1.34	0.123	43.7
Cross-sectional study	8	1.29	1.06–1.58	*0.013*	97.8
≥50 years	12	1.34	1.20–1.49	<*0.001*	87.0
Cohort study	4	1.37	1.16–1.60	<*0.001*	87.3
Cross-sectional study	8	1.27	1.15–1.40	<*0.001*	38.2
Passive smoking	4	1.13	1.09–1.16	<*0.001*	0
**Smoking cessation years**					
<10 years	4	1.09	0.96–1.25	0.170	42.1
≥10 years	4	0.93	0.79–1.10	0.413	41.0
**Smoking frequency**					
<10 cigarettes per day	3	1.08	1.01–1.15	*0.016*	0
10–20 cigarettes per day	3	1.06	1.01–1.12	*0.020*	0
≥20 cigarettes per day	2	1.41	0.86–2.31	0.176	89.0
**Smoking duration**					
<10 years	3	1.01	0.96–1.07	0.672	0
≥10 years	3	1.10	1.01–1.21	*0.034*	36.8
**Pack-years**					
<10	8	1.10	0.99–1.21	0.065	57.2
10–20	8	1.21	1.08–1.35	*0.001*	77.4
≥20	8	1.32	1.18–1.49	<*0.001*	85.4

OR, odds ratio; CI, confidence interval; NAFLD, non-alcoholic fatty liver disease; TC, total cholesterol; TG, triglyceride; HDL-C, high density lipoprotein cholesterol; LDL-C, low density lipoprotein cholesterol; FPG, fasting plasma glucose; SBP, systolic blood pressure; DBP, diastolic blood pressure; BMI, body mass index. The italics indicate significant differences in subgroup analysis (*p* < 0.05).

Regarding smoking history, ever smokers had a moderately elevated NAFLD risk (OR = 1.16, 95% CI: 1.06–1.28, *p* = 0.002), while former smokers also showed an increased risk (OR = 1.30, 95% CI: 1.05–1.60, *p* = 0.015). Notably, current smoking was more strongly associated with NAFLD (OR = 1.41, 95% CI: 1.22–1.63, *p* < 0.001), particularly in cohort studies (OR = 1.48, 95% CI: 1.25–1.75, *p* < 0.001), where heterogeneity was low (I^2^ = 19.1%) (Table 2).

### Subgroups analysis

Stratified analyses by metabolic indicators further demonstrated that the effect of smoking was more pronounced among individuals with elevated total cholesterol (≥5 mmol/L, OR = 1.47, 95% CI: 1.15–1.90, *p* = 0.003), triglycerides (≥1.2 mmol/L, OR = 1.41, 95% CI: 1.13–1.77, *p* = 0.003), or lower HDL-C (<1.30 mmol/L, OR = 1.52, 95% CI: 1.20–1.91, *p* < 0.001). Similar patterns were observed in participants with higher fasting plasma glucose (≥5.25 mmol/L, OR = 1.40, 95% CI: 1.18–1.66, *p* < 0.001) and elevated systolic blood pressure (≥125 mmHg, OR = 1.65, 95% CI: 1.41–1.93, *p* < 0.001) or diastolic blood pressure (≥75 mmHg, OR = 1.50, 95% CI: 1.05–2.14, *p* = 0.025) (Table 2).

Body mass index (BMI) significantly modified the association: among participants with BMI ≥ 24, the association was stronger (OR = 1.43, 95% CI: 1.20–1.71, *p* < 0.001) compared to those with BMI < 23.9 (OR = 1.21, 95% CI: 1.08–1.34, *p* = 0.001). The trend was more robust in cohort studies with high BMI (OR = 1.58, 95% CI: 1.35–1.85, *p* < 0.001) compared to those with low BMI (OR = 1.07, 95% CI: 0.98–1.17, *p* = 0.110) (Table 2).

In age subgroup analysis, older adults demonstrated a significant association (OR = 1.34, 95% CI: 1.20–1.49, *p* < 0.001), consistent across study designs (cohort study: OR = 1.37, 95% CI: 1.16–1.60, *p* < 0.001; cross-sectional study: OR = 1.27, 95% CI: 1.15–1.40, *p* < 0.001) (Table 2).

### Dose-response and passive exposure

A dose-response relationship between smoking and NAFLD was evident. Smoking ≥ 20 cigarettes per day was associated with a higher risk (OR = 1.41, 95% CI: 1.86–2.31, *p* = 0.176) compared to those with low smoking frequency (<10 cigarettes per day: OR = 1.08, 95% CI: 1.01–1.15, *p* = 0.016; 10–20 cigarettes per day: OR = 1.06, 95% CI: 1.01–1.12, *p* = 0.020), though with wide confidence intervals, indicating heterogeneity (I^2^ = 89.0%). Longer smoking duration (≥10 years) also conferred increased risk (OR = 1.10, 95% CI: 1.01–1.21, *p* = 0.034), while shorter duration showed no significant association. A similar trend was observed for pack-years, with stronger associations observed in participants with ≥20 pack-years (OR = 1.32, 95% CI: 1.18–1.49, *p* < 0.001) (Table 2).

In contrast, passive smoking was associated with a modest but statistically significant increase in NAFLD risk (OR = 1.13, 95% CI: 1.09–1.16, *p* < 0.001), with no observed heterogeneity (I^2^ = 0%), suggesting a consistent effect across studies (Table 2).

Moreover, the impact of smoking cessation varied by time since quitting. Those who had quit for less than 10 years still exhibited a non-significant increase in risk (OR = 1.09, 95% CI: 0.96–1.25, *p* = 0.170), while the risk was attenuated after ≥10 years of cessation (OR = 0.93, 95% CI: 0.79–1.10, *p* = 0.413) (Table 2).

## Discussion

This meta-analysis synthesizes findings from 19 observational studies, encompassing over 400,000 participants across multiple countries, to systematically evaluate the association between smoking and the risk of developing NAFLD. The pooled analysis demonstrated a significantly elevated risk of NAFLD among smokers, with current smokers exhibiting a higher risk compared to former and ever smokers. We specifically incorporated and stratified analyses by metabolic status (e.g., obesity, hypertension, dyslipidemia), allowing identification of high-risk subgroups and clarifying effect modification by metabolic health. These findings suggest that smoking may represent a critical modifiable risk factor for NAFLD.

Active smokers exhibited a significantly elevated risk of NAFLD, with this association remaining consistent across various study designs, including both cohort and cross-sectional analyses. Notably, both former and current smoking, as well as passive smoking, demonstrated significant correlations with NAFLD, suggesting persistent and potentially cumulative detrimental effects of tobacco exposure on hepatic function that may extend even years beyond smoking cessation (17). This is consistent with existing evidence indicating that the metabolic and inflammatory sequelae of tobacco use can persist over time, contributing to long-term hepatic steatosis and fibrosis. Stratified analyses further revealed that current smokers faced the highest NAFLD risk, implying that sustained and active exposure to cigarette smoke may exert more pronounced pathogenic effects, potentially via mechanisms such as oxidative stress, insulin resistance, lipid metabolism dysregulation, and pro-inflammatory cytokine activation (12, 18–21). The observed dose–response relationship, characterized by increasing NAFLD risk with longer duration and higher cumulative exposure to smoking, further supports a positive association between tobacco use and hepatic fat accumulation (22).

Importantly, passive smoking was also associated with a significantly increased risk of NAFLD, indicating that involuntary chronic exposure to environmental tobacco smoke may be sufficient to induce metabolic alterations and hepatocellular injury in non-smokers (23). Although the observed pooled effect size for the association between passive smoking and NAFLD incidence may appear modest at the individual level, its public health implications could be substantial. Given the vast number of individuals exposed to secondhand smoke worldwide, even a small increase in relative risk translates to a significant population attributable risk, suggesting that a considerable number of NAFLD cases could be potentially prevented through public health interventions aimed at reducing secondhand smoke exposure. This highlights the broader public health implications of secondhand smoke and underscores the need to address environmental exposure in NAFLD prevention strategies. The observed gradient of NAFLD risk–from highest in current smokers, intermediate in former smokers, and elevated in passive smokers compared to never-smokers–reinforces the importance of both primary and secondary prevention efforts (24). These findings emphasize the necessity of incorporating structured smoking cessation programs into NAFLD clinical management pathways and support the broader implementation of population-level tobacco-control policies to mitigate exposure in both private and public settings. Future studies should aim to elucidate the mechanistic pathways linking tobacco exposure to NAFLD progression and explore the reversibility of hepatic changes following smoking cessation (25, 26).

This study found that individuals who have been quitting smoking for at least 10 years have a significantly lower risk of developing NAFLD compared to current smokers. The mainstream views currently recognized by researchers are as follows: firstly, quitting smoking can alleviate oxidative stress and inflammation caused by tobacco smoke. Nicotine, tar, free radicals, and other substances in tobacco smoke are powerful pro-oxidants and pro-inflammatory agents (27). They directly reach the liver through the portal vein system or indirectly affect the liver by triggering systemic low-grade inflammation, exacerbating oxidative stress and inflammatory reactions in liver cells, which is the core link in the occurrence and development of NAFLD (28). After long-term smoking cessation, the body is freed from continuous exposure to exogenous toxins, and the antioxidant defense system (such as glutathione levels) in the body is restored. The levels of systemic inflammatory markers (such as C-reactive protein, TNF-α, IL-6) continue to decrease, creating a healthier metabolic environment for the liver (29). The second is to regulate the gut microbiota and gut liver axis (30). Studies have shown that smoking can alter the structure and function of the gut microbiota (dysbiosis), increase intestinal permeability, and lead to the translocation of endotoxins (such as lipopolysaccharides) to the portal vein. lipopolysaccharides exacerbate liver inflammation by activating immune cells in the liver (31). Long term smoking cessation can help restore the gut microbiota ecology and repair the intestinal barrier function, reducing the sustained attack on the liver (32). The third is to improve insulin resistance, restore insulin sensitivity, correct lipid metabolism, and reduce liver fat accumulation (33). The reversal of the above mechanism requires a long-term process, which may explain why it takes more than 10 years of continuous smoking cessation to observe a significant reduction in NAFLD risk, reflecting the time required for deep body repair. This evidence greatly enhances the scientific persuasiveness of smoking cessation initiatives. We suggest that future clinical guidelines and public health policies should fully consider reducing the risk of NAFLD as an important benefit in encouraging long-term smoking cessation and integrate it into patient education and public health information, in order to more effectively promote the overall liver health and overall health level of the population.

This study is the first to conduct comprehensive stratified analyses across subgroups defined by metabolic parameters such as blood pressure, BMI, and age, thereby providing novel insights into the interaction between smoking and metabolic health in the context of NAFLD (34–38). Notably, the magnitude of association between smoking and NAFLD was consistently more pronounced in these high-risk metabolic subgroups, implying that tobacco exposure may act synergistically with underlying metabolic dysregulation to accelerate hepatic steatosis and disease progression. This is biologically plausible, as smoking is known to impair lipid metabolism, exacerbate insulin resistance (38), and trigger systemic oxidative stress and inflammatory pathways (39) –all of which are established contributors to NAFLD pathogenesis. Particularly, populations with already compromised metabolic profiles, smoking may further destabilize hepatic lipid homeostasis, increase hepatic fat deposition, and heighten susceptibility to hepatocellular injury and fibrosis (40).

Although we adjusted for known confounding factors through a multivariate model, the possibility of residual confounding, such as confounding from measurement errors in diet, alcohol consumption, physical activity, and smoking, still exists. Especially, there is a high correlation between smoking and drinking behavior, which may partially contribute to the observed association. However, our sensitivity analysis indicates that a significant unmeasured confounding factor with a significant effect value is required to fully explain the current major findings, which reduces the likelihood of residual confounding leading to false positives. Therefore, although we interpret these findings cautiously, we believe that the observed associations are more likely to reflect the true effects rather than being solely due to confounding factors.

These findings underscore the importance of considering metabolic status when evaluating the hepatic impact of smoking and underscore the necessity for tailored preventive strategies in metabolically vulnerable populations (41). Public health interventions targeting smoking cessation may be particularly beneficial for individuals with existing cardiometabolic abnormalities, where the additive or synergistic effects of smoking may significantly increase the burden of liver disease (41, 42). Future mechanistic and longitudinal studies are warranted to further delineate these interactions and assess whether risk attenuation is achievable following cessation in these high-risk groups.

Given the marked increase in NAFLD risk associated with both active and passive smoking–particularly among people with obesity, dyslipidemia, or hypertension–smoking history should be routinely assessed in metabolically at-risk populations. Smoking cessation interventions should be incorporated into comprehensive NAFLD prevention and management protocols. At the public health level, enhancing tobacco control policies and raising awareness of the liver-specific risks of smoking could help mitigate the growing global burden of NAFLD. Despite the strength of the evidence, several limitations must be acknowledged. First, although the overall sample size is large, certain subgroup analyses may have been underpowered due to limited primary studies, which could affect the precision of risk estimates in these categories. Second, significant heterogeneity was observed across included studies, likely attributable to variations in NAFLD diagnostic criteria, differences in participant characteristics across geographic regions, and diverse adjustments for confounding factors. Third, varying diagnostic methods for NAFLD (e.g., ultrasound vs. biomarkers) across studies may affect comparability. Differences in diagnostic methods and regional distribution of populations may be important reasons for heterogeneity. The fourth is the limitation of exposure assessment. The main research relies on self-reported questionnaires to determine passive smoking exposure, which is susceptible to recall and misclassification bias. Therefore, the risk estimation proposed in our study may underestimate the true extent of the association between passive smoking and NAFLD. It is necessary to conduct prospective studies using objective exposure biomarkers (such as serum cotinine levels) in the future to confirm our findings and provide more accurate risk assessments.

This study and the cited literature both use the traditional term “NAFLD.” However, during the course of this study, the International Liver Disease Expert Group issued a new consensus recommendation to update the terminology to “metabolic dysfunction associated fatty liver disease (MASLD)” (43). Given that the diagnostic criteria for NAFLD included in the study (details in Supplementary Table 1) are highly consistent with those for MASLD, the clinical characteristics of the study population essentially overlap with the current definition of MASLD. Out of respect for the original research design and consistency of data sources, this article still uses the term “NAFLD,” but the results of this study are also applicable to patient populations that meet the diagnostic criteria for MASLD.

## Conclusion

This meta-analysis demonstrates a significant association between smoking and increased risk of NAFLD. Both current and former smokers are at elevated risk, with stronger associations seen in current smokers. These findings highlight the importance of smoking cessation in the prevention of NAFLD and reinforce public health efforts targeting lifestyle modifications to reduce liver-related morbidity. Future longitudinal studies with standardized NAFLD diagnostic criteria and detailed smoking exposure assessments are warranted to further elucidate causality and underlying mechanisms.

## Data Availability

The original contributions presented in this study are included in this article/Supplementary material, further inquiries can be directed to the corresponding author.
