# Supplementary material for: The relationship between tobacco and non-alcoholic fatty liver disease incidence: a systematic review and meta-analysis of observational studies
Source: Front Med (Lausanne). 2025 Oct 15;12:1670932. doi: 10.3389/fmed.2025.1670932 (PMC12568600; doi:10.3389/fmed.2025.1670932)
Supplement: Supplementary file 1 [file Table_1.docx]

**Supplementary Table 1**. Characteristics of included observational studies in the meta-analysis

| Author, year | Number of smokers (include active and passive) | Number of non-smokers | Median follow up time (years) | Study name | Diagnostic methods for NAFLD | Exposure assessment | Adjusting parameters |
| --- | --- | --- | --- | --- | --- | --- | --- |
| Llorenc¸ Caballerı´a, 2009 | 342 | 424 | NA | NA | the diagnosis of NAFLD was achieved by abdominal echography according to standard criteria.The grade of involvement was standardized using a semiquantitative scale of the grade of hepatic enhancement. | interview | age, sex and alcohol consumption |
| Ayaka Hamabe, 2001 | 445 | NA | NA | NA | abdominal ultrasonography was performed by trained technicians. Fatty liver was diagnosed when hepatorenal echo contrast and liver brightness were observed. | questionnaire | age, sex, obesity, hypertension, dyslipidemia, dysglycemia and alcohol intake |
| Edith M. Koehler, 2012 | 1782 | 1029 | NA | The Population-based Rotterdam Study | abdominal ultrasonography was performed by certified and experienced technicians on Hitachi HI VISION 900. Severity of fatty liver was classified as "no fatty liver" (score 0–1), "mild fatty liver" (score 2–3), or "moderate to severe fatty liver" (score 4–6). | questionnaire | age, gender, educational level, pack years of smoking, total physical activity level and alcohol consumption |
| Yu Liu, 2013 | 4975 | 999 | NA | NA | a diagnosis of fatty liver was based on the presence of at least 2 of the following 3 abnormal findings: diffuse increased echogenicity of liver relative to kidney, ultrasound beam attenuation, and poor visualization of intrahepatic structures. | interview | age, education status, alcohol consumption, physical activity, obesity, hypertension, diabetes,  use of antidiabetic medication, and dyslipidemia |
| Eline H. van den Berg, 2017 | 7008 | NA | NA | The Lifelines Cohort Study | for the diagnosis of NAFLD the algorithm of the fatty liver index (FLI) was used. | questionnaire | age, sex, hemoglobin, albumin, platelets, cholesterol, total leucocytes, cardiovascular disease, impaired renal function |
| Peiyi Liu, 2017 | 5607 | 3825 | NA | The Dongfeng-Tongji Cohort Study | fatty liver was diagnosed in the presence of diffuse increase of hepatic echogenicity and decrease in density, accompanying unclear display of intra-hepatic lacuna structure or mild to moderate hepatomegaly with a round and blunt border. | interview | age, BMI, waist and diabetes |
| Nam Hee Kim, 2017 | 52915 | 107947 | NA | The Kangbuk Samsung Health Study | fatty liver was diagnosed and graded as normal, mild, moderate, or severe hepatic steatosis on the basis of the following four known criteria: hepatorenal echogenic contrast, liver brightness, deep attenuation, and vascular blurring. | questionnaire | age, sex, obesity, alcohol use, physical activity, and educational level |
| Masashi Okamoto, 2018 | 560 | 3300 | 3.96 | NA | fatty liver disease was diagnosed based on the following established diagnostic criteria: hepatorenal echo contrast, liver brightfness, deep attenuation, and vascular blurring. | questionnaire | age, gender, the body mass index , the systolic blood pressure, the total cholesterol, triacylglycerols, fasting plasma glucose, uric acid, creatinine levels, the exercise and snacking habits, the sleep duration, and the alcohol consumption status |
| Xianghai Zhou, 2019 | 951 | 2215 | NA | NA | NAFLD was defined using a liver‐spleen ratio≤1.1 on unenhanced abdominal computed tomography scanning. | questionnaire | age, BMI, systolic blood pressure, total cholesterol, triglycerides, uric acid, annual house income, education level, physical activity level, smoking status (for men), red meat  intake, and alcohol consumption |
| Julianna C Hsing, 2019 | 637 | 2952 | 2.3 | The Wellness Living Laboratory China Study | “Yes NAFLD” (FLI >60), “Maybe NAFLD” (FLI 30-60), and “No NAFLD” (FLI <30). | questionnaire | age, sex, and income, and insulin resistance |
| Haruka Takenaka, 2020 | 3746 | 9720 | 30.2 | NA | fatty liver was diagnosed based on the abdominal ultrasonography findings when hepatorenal echo contrast and liver brightness were observed. | questionnaire | age, sex, presence of metabolic syndrome and light alcohol consumption |
| Feitong Wu, 2021 | NA | NA | 31 | The Cardiovascular Risk In Young Finns Study | fatty liver was assessed by ultrasound imaging of the liver using a validated protocol and Sequoia 512 ultrasound mainframes (Acuson, Mountain View, CA, USA) with 4.0 MHz adult abdominal transducers. | questionnaire | age, sex, child serum insulin (log-transformed), BMI and parental school years, and adult physical activity and alcohol consumption, adult mean BMI, adult waist circumference, systolic blood pressure, triglycerides, and insulin levels |
| Joon Ho Moon, 2021 | 7576 | 20483 | 6.8 | The Korean National Health And Nutrition Examination Surveys | NAFLD was defined as a hepatic steatosis index >36 | questionnaire | age, sex, and BMI |
| Seogsong Jeong, 2023 | 10611 | 128569 | 21.3 | The National Health Insurance Service-National Sample Cohort | the presence of NAFLD was operationally defined using the FLI. FLI ≥60 was defined as NAFLD. | questionnaire | age, sex, household income, BMI, hypertension, diabetes mellitus, dyslipidemia, and charlson comorbidity index |
| Haofei Hu, 2022 | 5505 | 8746 | 11.7 | NA | an abdominal ultrasound was used to assess NAFLD, and gastroenterologists without knowledge of the participants’  personal information, reviewed the ultrasound images. The  final diagnosis was made based on the evaluation of four  ultrasound findings: liver brightness, liver and kidney echo  contrast, vessel blurring, and depth attenuation. | questionnaire | age, systolic blood pressure, total  cholesterol, gamma-glutamyl transferase, diastolic  blood pressure, alanine amino transferase,  ethanol consumption, aspartate amino transferase,  hemoglobin A1c, the habit exercise, smoking status, and sex. |
| Paulina Pettinelli, 2023 | 1148 | 1148 | 4.3 | The National Health Survey Of Chile 2016–2017 | The FLI and the lipid accumulation product are two of these methods. | questionnaire | age, sex, BMI and education |
| Minjung Han, 2023 | 3980 | 3110 | 5 | The 7Th And 8Th Korea National Health And Nutrition  Examination Survey | NAFLD as determined by ultrasonography was used as the reference standard. | questionnaire | age, body BMI, overweight or obese, household income, education, employment status, alcohol consumption, physical activity, hypertension, dyslipidemia |
| Yun Seo Jang, 2023 | 3494 | 6109 | 2 | The 2019–2020  National Health And Nutrition Examination Survey | the NAFLD liver fat score  developed by the Department of Medicine and the Minerva  Medical Research Institute at Helsinki University. Participants were considered to have NAFLD if their liver fat score of NAFLD was > -0.640 as the optimal cutoff point. | questionnaire | demographic factors (sex, age, marital  status, and educational level), socioeconomic factors (household  income, region, and occupational categories), behavioral health  patterns (current drinking status, physical activity), and health_x005f related factors (BMI, diagnosis of hypertension, and diagnosis of diabetes) |
| Ying Che, 2023 | 814 | 10 | 1 | The Chinese Nurse Cohort Study | the diagnostic criteria of NAFLD in this study: there was no history of drinking alcohol or the alcohol equivalent amount was less than 70 g/week; diseases that can lead to fatty liver such as viral hepatitis, drug finduced liver disease, and autoimmune liver disease were excluded; imaging of diffuse hepatic steatosis. In this study, the results of abdominal B-ultrasound were used as imaging evidence. | questionnaire | NA |

NA: not available; NAFLD: non-alcoholic fatty liver disease; FLI: fatty liver index; BMI: body mass index;
