# Supplementary material for: The relationship between tobacco and non-alcoholic fatty liver disease incidence: a systematic review and meta-analysis of observational studies
Source: Front Med (Lausanne). 2025 Oct 15;12:1670932. doi: 10.3389/fmed.2025.1670932 (PMC12568600; doi:10.3389/fmed.2025.1670932)
Supplement: Supplementary file 2 [file Table_2.docx]

**Supplementary Table 2.** Quality assessment of cohort studies included.

| Author, year,  Study (Observational) | **Selection (Out of 4)** | | | | **Comparability**  **(Out of 2)** | **Outcomes (Out of 3)** | | | **Total**  **(Out of 9)** |
| --- | --- | --- | --- | --- | --- | --- | --- | --- | --- |
|  | Representativeness of exposed cohort | Selection of non exposed cohort | Ascertainment  of exposure | Outcome not present at the start of the study |  | Assessment of outcomes | Length of follow-up | Adequacy of follow up of cohorts |  |
| Ayaka Hamabe, 2001 | 1 | 1 | 1 | 1 | 1 | 1 | 0 | 1 | 7 |
| Edith M. Koehler, 2012 | 1 | 1 | 1 | 1 | 2 | 1 | 1 | 1 | 9 |
| Masashi Okamoto, 2018 | 1 | 1 | 1 | 1 | 1 | 0 | 1 | 1 | 7 |
| Feitong Wu, 2021 | 1 | 0 | 1 | 1 | 2 | 1 | 1 | 1 | 8 |
| Joon Ho Moon, 2021 | 1 | 1 | 1 | 1 | 2 | 1 | 1 | 1 | 9 |
| Seogsong Jeong, 2023 | 1 | 1 | 0 | 1 | 2 | 1 | 1 | 1 | 8 |
| Paulina Pettinelli, 2023 | 1 | 1 | 1 | 1 | 1 | 1 | 1 | 1 | 8 |
| Minjung Han, 2023 | 0 | 1 | 1 | 1 | 2 | 1 | 1 | 0 | 7 |
| Yun Seo Jang, 2023 | 1 | 1 | 1 | 1 | 1 | 1 | 0 | 1 | 7 |
| Ying Che, 2023 | 1 | 1 | 1 | 1 | 2 | 1 | 1 | 1 | 9 |

The observational studies were assessed by the Newcastle-Ottawa Quality Assessment Scale (NOS) checklist of cohort studies.
