# Supplementary material for: The relationship between tobacco and non-alcoholic fatty liver disease incidence: a systematic review and meta-analysis of observational studies
Source: Front Med (Lausanne). 2025 Oct 15;12:1670932. doi: 10.3389/fmed.2025.1670932 (PMC12568600; doi:10.3389/fmed.2025.1670932)
Supplement: Supplementary file 3 [file Table_3.docx]

**Supplementary Table 3.** Quality assessment of case control studies included.

| Author, year,  Study (Observational) | **Selection (Out of 4)** | | | | **Comparability**  **(Out of 2)** | **Outcomes (Out of 3)** | | | **Total**  **(Out of 9)** |
| --- | --- | --- | --- | --- | --- | --- | --- | --- | --- |
|  | Adequate case definition | Representativeness of the cases | Selection of controls | Definition of controls |  | Ascertainment of exposure | Same method of ascertainment for cases and controls | Non-response rate |  |
| Haruka Takenaka, 2020 | 1 | 1 | 1 | 1 | 2 | 1 | 1 | 1 | 9 |

The observational studies were assessed by the Newcastle-Ottawa Quality Assessment Scale (NOS) checklist of case control studies.
