# Supplementary material for: The relationship between tobacco and non-alcoholic fatty liver disease incidence: a systematic review and meta-analysis of observational studies
Source: Front Med (Lausanne). 2025 Oct 15;12:1670932. doi: 10.3389/fmed.2025.1670932 (PMC12568600; doi:10.3389/fmed.2025.1670932)
Supplement: Supplementary file 4 [file Table_4.docx]

**Supplementary Table 4.** Quality assessment of cross-sectional studies included.

| Author, year  Study (Cross-Sectional) | Information source | Inclusion and exclusion criteria | Indicate time period | Consecutive subjects | Evaluation of subjective components | Ascertainment of exposure | Explain for exclusions | Control confounding | Missing data | Completeness of data collection | Incomplete data or follow-up |
| --- | --- | --- | --- | --- | --- | --- | --- | --- | --- | --- | --- |
| Llorenç Caballería, 2009 | low risk | low risk | low risk | unclear risk | low risk | low risk | low risk | unclear risk | unclear risk | low risk | low risk |
| Yu Liu, 2013 | low risk | low risk | unclear risk | low risk | unclear risk | low risk | low risk | low risk | unclear risk | unclear risk | low risk |
| Eline H. van den Berg, 2017 | low risk | low risk | low risk | unclear risk | low risk | unclear risk | low risk | unclear risk | low risk | low risk | unclear risk |
| Peiyi Liu, 2017 | low risk | low risk | low risk | unclear risk | unclear risk | low risk | low risk | high risk | low risk | low risk | low risk |
| Nam Hee Kim, 2017 | low risk | low risk | low risk | low risk | low risk | unclear risk | low risk | low risk | low risk | low risk | low risk |
| Xianghai Zhou, 2019 | low risk | low risk | low risk | high risk | unclear risk | low risk | low risk | unclear risk | low risk | unclear risk | low risk |
| Julianna C Hsing, 2019 | low risk | unclear risk | unclear risk | low risk | low risk | low risk | low risk | low risk | low risk | low risk | low risk |
| Haofei Hu, 2022 | low risk | low risk | low risk | unclear risk | low risk | unclear risk | low risk | unclear risk | low risk | low risk | unclear risk |

The cross-sectional studies were assessed by the Agency for Healthcare Research and Quality (AHRQ) Recommended Standard List.

Risk of bias was assessed as “low risk”, “high risk” or “unclear risk”.
